# [Expression of Concern] MicroRNA-221 regulates cell activity and apoptosis in acute lymphoblastic leukemia via regulating PTEN

**DOI:** 10.3892/etm.2026.13255

**Published:** 2026-07-29

**Authors:** Lingyan Zhang, Zibin Bu, Juan Shen, Liping Shang, Yuanyuan Chen, Ping Zhang, Yan Wang

Exp Ther Med 22:1133, 2021; DOI: 10.3892/etm.2021.10567

Following the publication of the above paper, it was drawn to the Editor’s attention by a concerned reader that the expression of BAX protein in Jurkat T cells, as shown in the western blots in Fig. 1G on p. 3, was unexpected, since Jurkat cells have been reported to be BAX-null [see the paper “A genome-wide survey of mutations in the Jurkat cell line” by Gioia *et al*, Vol. 19, article no. 334 (2018)].

The authors have been contacted by the Editorial Office to offer an explanation for the apparent anomaly in the presentation of the data in this paper, although up to this time, no response from them has been forthcoming. Owing to the fact that the Editorial Office has been made aware of potential issues surrounding the scientific integrity of this paper, we are issuing an Expression of Concern to notify readers of this potential problem while the Editorial Office continues to investigate this matter further.

